# Neuroprotective Properties of Berberine: Molecular Mechanisms and Clinical Implications

**DOI:** 10.3390/antiox12101883

**Published:** 2023-10-19

**Authors:** Erjie Tian, Gaurav Sharma, Chongshan Dai

**Affiliations:** 1 College of Animal Science and Technology, Henan University of Science and Technology, Kaiyuan Avenue 263, Luoyang 471000, China; 2 Cardiovascular and Thoracic Surgery and Advanced Imaging Research Center, University of Texas Southwestern Medical Center, Dallas, TX 75230, USA; 3 National Key Laboratory of Veterinary Public Health and Safety, College of Veterinary Medicine, China Agricultural University, Beijing 100193, China; 4 Key Biology Laboratory of Chinese Veterinary Medicine, Ministry of Agriculture and Rural Affairs, Beijing 100193, China

**Keywords:** berberine, neuroprotective effects, molecular mechanisms, clinical applications

## Abstract

Berberine (BBR), an isoquinoline alkaloid natural product, is isolated primarily from *Coptis chinensis* and other *Berberis* plants. BBR possesses various bioactivities, including antioxidant, anti-inflammation, anticancer, immune-regulation, and antimicrobial activities. Growing scientific evidence underscores BBR’s substantial neuroprotective potential, prompting increased interest and scrutiny. In this comprehensive review, we elucidate the neuroprotective attributes of BBR, delineate the underlying molecular mechanisms, and assess its clinical safety and efficacy. The multifaceted molecular mechanisms responsible for BBR’s neuroprotection encompass the attenuation of oxidative stress, mitigation of inflammatory responses, inhibition of apoptotic pathways, facilitation of autophagic processes, and modulation of CYP450 enzyme activities, neurotransmitter levels, and gut microbiota composition. Furthermore, BBR engages numerous signaling pathways, including the PI3K/Akt, NF-κB, AMPK, CREB, Nrf2, and MAPK pathways, to confer its neuroprotective effects. This comprehensive review aims to provide a substantial knowledge base, stimulate broader scientific discourse, and facilitate advancements in the application of BBR for neuroprotection.

## 1. Introduction

**Figure 1:**
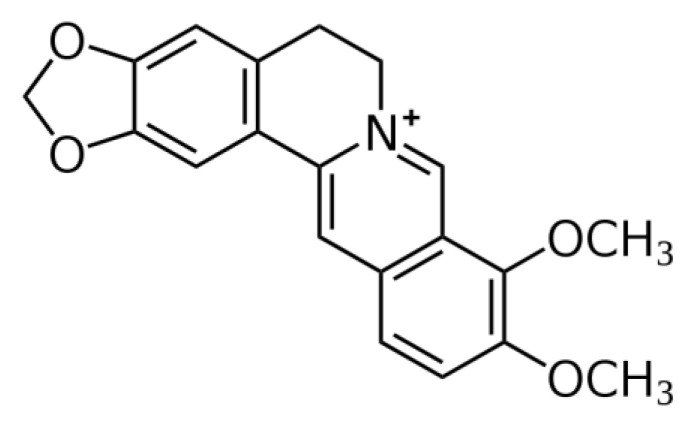
Chemical structure of BBR.

Berberine (BBR), a quaternary isoquinoline alkaloid compound, has been identified in several plants, including *Ranunculus canadensis*, *Berberis vulgaris*, *Berberis aquifolium*, and *Coptis chinensis* [1,2,3]. BBR is a yellow acicular crystal with the molecular formula of C_20_H_18_NO_4_, and its chemical structure is shown in Figure 1. Its current clinical use is often in the form of hydrochloride (i.e., BBR hydrochloride) or sulfate (i.e., BBR sulfate). It is well known that BBR has a series of pharmacological activities, including antimicrobial, antioxidant, anti-inflammatory, anti-heart failure, anti-arrhythmia, lowering cholesterol, anticancer, and immunomodulatory activities [4,5,6,7,8,9,10]. In China, the China Food and Drug Administration has approved BBR for treating bacterial-induced intestinal diarrhea in both humans and livestock. Despite historically limited bioavailability, ongoing preclinical trials are driving exploration into new potential applications [11,12].

Recent research has revealed BBR’s ability to cross the blood–brain barrier, yielding positive impacts on brain functions [13,14,15,16,17,18,19,20]. Numerous in vitro and animal studies have showcased BBR’s capacity to offer neuroprotection against drug- and toxin-induced neurotoxicity, ischemia–reperfusion injury, and chronic neurodegenerative conditions such as Alzheimer’s, Parkinson’s, and Huntington’s diseases [13,14,15,16,17,18,19,20]. The intricate molecular mechanisms underlying BBR’s robust neuroprotective effects encompass various biological functions, including antioxidative, anti-inflammatory, and antiapoptotic actions [21]. For this review, the most important articles published from 1 January 2000 to 1 August 2023 relating to the protective effects of BBR on neurological diseases were selected from the Scopus, PubMed, and Web of Science databases. The keyword combinations used for the literature search include: ‘berberine and neuroprotection’, ‘berberine and neuroprotection’, ‘berberine and neuroprotective effect’, or ‘berberine and neurodegenerative diseases’. In addition, some important studies involving the side effects and toxic doses of BBR were also selected. In this present review, we aimed to summarize the neuroprotective effects, molecular mechanisms, side effects and toxic dosages, and clinical applications of BBR while also addressing existing challenges associated with its use as a neuroprotective agent.

## 2. An Overview of the Neuroprotective Effects of BBR

BBR possesses a remarkable capacity to cross the blood–brain barrier (BBB) and confer potent neuroprotection against a spectrum of neurodegenerative conditions, including Alzheimer’s disease, Parkinson’s disease, cerebral ischemia, mental depression, schizophrenia, and anxiety [5,8,19,22,23,24]. It has been reported that oral administration of BBR could effectively treat neurotoxic injury caused by drugs (e.g., doxorubicin, and 6-hydroxydopamine [6-OHDA]), environmental toxins (e.g., chlorpyrifos, mercury, aluminum, cadmium, and fluoride), aging (e.g., amyloid β-induced aging), ischemia–reperfusion, or stroke (i.e., middle cerebral artery occlusion-induced stroke) both in vivo and in vitro [15,25,26,27,28,29].

Furthermore, advancements in nanotechnology and nose-to-brain drug delivery (NBDD) techniques have enhanced BBR’s ability to penetrate the blood–brain barrier and achieve targeted brain delivery [30,31,32,33]. For instance, BBR-loaded nanostructured lipid carriers coated with chitosan (BBR-CTS-NLCs) have demonstrated improved brain targeting and enhanced therapeutic efficacy in the treatment of central nervous system diseases, such as Alzheimer’s disease, through nasal administration [34].

The molecular mechanisms of BBR’s neuroprotection are multifaceted and context-dependent. Over the past three decades, researchers have demonstrated that the neuroprotective mechanisms of BBR involve the inhibition of oxidative stress, mitochondrial dysfunction, inflammatory response, programmatic cell death (e.g., ferroptosis, necroptosis, and apoptosis), and the activation of autophagy [8,15,25,26,27,28,29,35,36]. Several signaling pathways, including phosphoinositide 3-kinase (PI3K)/protein kinase B (Akt), mitogen-activated protein kinases (MAPKs), AMP-activated protein kinase (AMPK), hypoxia-inducible factor-1 (HIF-1), autophagy, nuclear factor kappa B (NF-κB), peroxisome proliferator-activated receptors (PPARs), cyclic AMP response element (CRE)-binding protein (CREB), p53, nuclear factor-E2-related factor 2 (Nrf2), and mitochondrial apoptotic pathways, were also involved [8,15,25,26,27,28,29,35,36]. Recent investigations have even indicated that oral BBR supplementation can elevate brain dopamine (DA) levels, ameliorating Parkinson’s disease by modulating gut microbiota, adding another layer of complexity to its mechanisms of action [37]. The detailed mechanistic aspects will be expounded upon in subsequent sections.

## 3. Molecular Mechanisms of BBR’s Neuroprotection

### 3.1. Inhibition of Oxidative Stress, Mitochondrial Dysfunction, and Apoptosis

Growing evidence underscores the pivotal role of oxidative stress in the initiation and progression of neurodegenerative disorders [38,39,40,41,42,43,44,45,46,47,48,49]. Excessive production of reactive oxygen species (ROS) and reactive nitrogen species (RNS) under oxidative stress conditions can induce lipid peroxidation, protein oxidation, protein nitration, and glycol oxidation, culminating in membrane damage, cytoskeletal abnormalities, and DNA damage within neural tissues [38,39,40,41,42,43,44,45,46,47,48,49]. Consequently, antioxidant supplementation has emerged as an effective therapeutic strategy for neurodegenerative diseases [50,51,52,53].

Numerous studies have shed light on the potent antioxidant properties of BBR, making it a promising option for treating neurodegenerative diseases [6,8,14,54,55,56,57,58,59,60,61,62,63,64,65,66,67]. In vitro investigations have demonstrated its ability to scavenge peroxynitrite (ONOO^−^), nitric oxide (NO), hydroxyl radical (OH^•^), superoxide anion (O_2_^•−^), and sodium nitroprusside, cisplatin, and Fe^2+^-induced lipid peroxidation (LPO) [68]. Using in vitro 2, 2-diphenyl-1-picrylhydrazyl radical (DPPH) and 2, 2-azinobis (3-ethylbenzothiazoline-6-sulfonate) radical tests showed that the half-maximal inhibitory concentrations (IC_50_s) of BBR are both about 0.3 mg/mL [69]. In addition, it was also found that the IC_50_s of BBR for NO radical scavenging, Fe^2+^ chelation, and OH^•^ radical scavenging are 0.17 mg/mL, 0.12 mg/mL, and 0.11 mg/mL, respectively [69]. Notably, ONOO^−^ is a potent oxidative and nitrating reagent capable of damaging various intracellular macromolecules, including proteins, lipids, and DNA. BBR effectively reduces superoxide levels in macrophages mediated by NADPH oxidase, restoring cellular redox balance by selectively inhibiting gp91phox expression and enhancing antioxidant enzyme activities, such as superoxide dismutase (SOD) and catalase (CAT), thereby mitigating oxidative-stress-induced cytotoxicity and brain injury [55,70]. Animal studies have corroborated these findings, showing that BBR supplementation significantly reduces malondialdehyde (MDA) levels, enhances SOD and CAT activities, and inhibits caspase activities in the hippocampus tissues of rats, thereby attenuating neurodegeneration induced by two-vessel occlusion [61]. Additionally, BBR has been shown to prevent mitochondrial ROS generation by targeting the N-methyl-D-aspartate-receptor (NMDA)R1/NADPH oxidase 3 (NOX3) pathway, protecting spiral ganglion cells from cytomegalovirus-induced apoptosis [71].

Mitochondria, being both the main targets and producers of ROS, play a central role in oxidative-stress-related neurodegeneration [72]. Studies have demonstrated that ROS production is regulated by various antioxidant defense pathways, including the PI3K/Akt pathway, Nrf2 pathway, and PPAR pathway [24,73,74]. *Nrf2*, a key regulator of antioxidant defense, transcriptionally controls the expression of numerous protective genes in response to oxidative stress [75]. Although direct interactions between BBR and *Nrf2* require further investigation, studies have shown that BBR supplementation activates *Nrf2* and its downstream target, heme oxygenase-1 (*HO-1*), exerting neuroprotective effects [75]. Many studies have reported that the *Nrf2*–antioxidant response element axis is a critical target against oxidative stress in neurodegenerative diseases [76]. Albeit it still lacks the direct evidence of BBR interaction with *Nrf2*, some studies have illustrated that BBR supplementation could activate the expression of *Nrf2* and its downstream gene *HO-1*, exhibiting its neuroprotective effects [24]. Activation of the Akt signaling pathway by BBR inhibits ROS production, protecting against oxidative stress induced by various agents [77,78]. Consistently, it has been reported that BBR supplementation could alleviate rotenone-induced ROS production in human neuroblastoma cells (i.e., SH-SY5Y cells) via the inhibition of mitochondrial dysfunction through activating the PI3K/Akt signaling pathway [66]. Moreover, BBR has been reported to alleviate rotenone-induced ROS production via the activation of *Nrf2* and *HO-1* expression, safeguarding against high-glucose-induced cell apoptosis [79]. Hsu et al. demonstrated that BBR activates Nrf2 nuclear translocation and protects NSC34 motor neuron-like cells from oxidative damage through the activation of PI3K/Akt-dependent cytoprotective pathways [80]. Recent findings highlight BBR as a potent ligand of PPARδ, promoting Nrf2 and NQO1 expression and consequently mitigating oxidative stress and brain injury in a mouse model of middle cerebral artery occlusion (MCAO) [74]. In short, the antioxidant defense function of BBR against neurotoxic effects caused by oxidative stress mainly depends on its free radical scavenging ability and the activation of endogenous antioxidant signaling pathways, such as the Nrf2, PI3K/Akt, and PPARδ pathways.

Mitochondrial dysfunction, stemming from excessive reactive oxygen species (ROS) production, triggers a cascade of events, including ATP depletion, the opening of mitochondrial permeability transition pores, caspase activation, and cellular apoptosis [81]. The opening of mitochondrial permeability transition pores is regulated by various proteins and signaling networks, including mitochondrial membrane potential, mitochondrial Ca^2+^ signals, and members of the Bcl-2 family (e.g., the antiapoptotic B-cell lymphoma-extra-large [Bcl-XL] and Bcl-2; the proapoptotic members of the family: Bcl-2-associated X protein [BAX], Bcl-2 antagonist/killer 1 [BAK1], and Bcl-2-associated agonist of cell death [BAD]) [82]. BBR supplementation has been shown to upregulate mitochondrial membrane potential and ATP levels, protecting against amyloid-β-induced mitochondrial dysfunction and cell apoptosis in primary cultured hippocampal neurons [83]. BBR inhibits the release of proapoptotic factors like cytochrome c and apoptosis-inducing factors (AIFs) in response to oxygen–glucose deprivation (OGD), safeguarding against ischemic brain injury [84]. In a rat model, Singh et al. found that oral BBR supplementation at the doses of 10 or 20 mg/kg/day for 19 days could significantly improve the mitochondrial complex (I, II, and IV) activities and inhibit the activation of caspase-3 in brain tissues, followed by the amelioration of cerebral ischemia-induced brain injury [85]. The neuroprotective effect of BBR against ischemia-induced brain injury involves the activation of the Akt/GSK3β/ERK1/2 signal pathway and the inhiation of the JNK/caspase-3 pathway [18]. In addition, oral BBR supplementation could upregulate the expression of Bcl-2 protein and downregulate the expression of Bax protein in the brain tissue, inhibiting cell apoptosis and ameliorating doxorubicin-induced cognitive impairment in rats [26]. Zhang et al. found that BBR pretreatment attenuated hypoxia condition-induced neuronal cell death or brain injury through the downregulation of HIF-1α protein, inhibiting caspase-9 and caspase-3 activations and decreasing the Bcl-2/Bax ratio in PC12 cells in a rat model [86].

**Figure 2:**
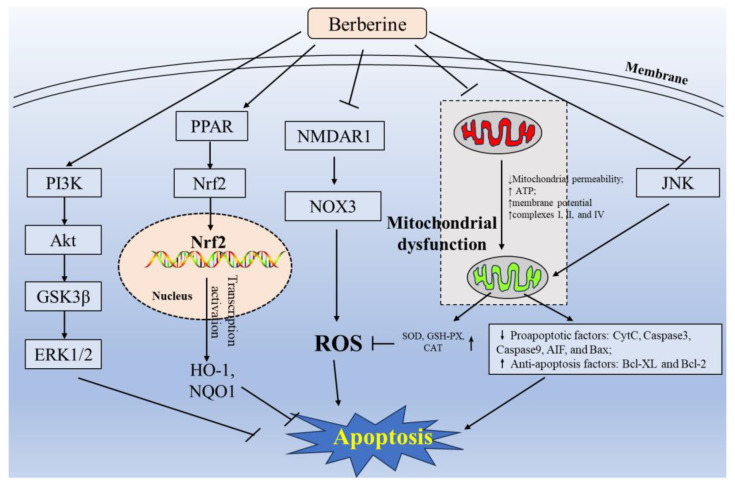
A proposed mechanism of neuroprotection by BBR against oxidative stress, mitochondrial dysfunction, and apoptosis. ↑ indicates the upregulation by BBR; ↓ indicates the downregulation by BBR. PI3K, phosphoinositide 3-kinase; Akt, protein kinase B; GSK3β, glycogen synthase kinase 3β; ERK1/2, extracellular regulated protein kinases 1/2; PPAR, peroxisome proliferators-activated receptor; Nrf2, nuclear factor-E2-related factor 2; HO-1, heme oxygenase-1; NQO1, NAD (P)H quinone oxidoreductase 1; NMDAR1, N-methyl-d-aspartate receptor 1; NOX3, NADPH oxidase 3; ROS, reactive oxygen species; JNK, c-Jun N-terminal kinase; ATP, adenosine triphosphate; SOD, superoxide dismutase; GSH-PX, glutathione peroxidase; CAT, catalase; CytC, cytochrome c; AIF, apoptosis-inducing factor; Bax, Bcl-2-associated X protein; Bcl2, B-cell lymphoma-2; Bcl-XL, B-cell lymphoma-extra-large.

Taken together, as shown in Figure 2, BBR exerts its neuroprotective effects by inhibiting ROS production, mitochondrial apoptotic pathways, and neuronal cell apoptosis through the activation of various signaling pathways, including PI3K/Akt, PPAR/Nrf2, NMDAR1/NOX3, JNK, and the regulation of mitochondrial function.

### 3.2. Blockade of Inflammatory Response and Necroptosis

Previous studies have convincingly established that mitigating neuroinflammation could mitigate neuronal loss and decrease the morbidity associated with neurodegenerative disorders [35,87,88,89]. Neuroinflammation, characterized by the chronic activation of microglia and astrocytes, can result from various factors such as traumatic brain injury, microbial infection, drugs, neurotoxins, or toxic metabolites [35,87,88,89]. In the context of neurodegenerative diseases, the activation of microglia and astrocytes often leads to the secretion of multiple proinflammatory mediators and neurotoxic cytokines. This, in turn, fuels a detrimental cycle of neuronal damage and neuroinflammation, ultimately driving the chronic progression of neurodegenerative conditions [35,87,88,89].

Berberine (BBR) stands out as a promising agent for combating neuroinflammation, effectively regulating the inflammatory response triggered by infections, toxins, aging, or ischemia–reperfusion through multiple signaling pathways [23,90,91,92,93]. In an in vitro model simulating the pathology of Alzheimer’s disease, BBR alleviates neuroinflammatory response by reducing the production of proinflammatory cytokines in microglia [94]. Furthermore, BBR partially ameliorates cognitive dysfunction induced by lipopolysaccharide (LPS) by attenuating neuroinflammation [95]. Jia et al. found that BBR supplementation could inhibit Aβ-induced microglia inflammation by suppressing the activation of the NF-κB and MAPK signaling pathways [96]. Zhang et al. found that BBR could reduce ischemic brain injury and neuroinflammatory response via increasing the activation of the Akt/glycogen synthase kinase (GSK) signaling pathway and inhibiting the NF-κB pathway [97]. BBR supplementation could reduce TLR4/MyD88/NF-κB signaling transduction and attenuate neuronal death induced by microglial-conditioned media [92]. BBR effectively inhibits the inflammatory activation of rat brain microglia by inactivating the NF-κB/iNOS/NO pathway or activating the AMPK pathway [90,98,99]. Additionally, several studies have reported that BBR directly upregulates the expression of anti-inflammatory factors (e.g., interleukin (IL)-4 [IL-4] and IL-10) while inhibiting the expression of proinflammatory factors (e.g., COX-2, prostaglandin E2 [PGE2]), IL-1β, IL-6, and tumor necrosis factor-α (TNF-α)), displaying therapeutic effects against neuroinflammatory diseases in both in vitro and in vivo settings [99,100,101,102].

Necroptosis, a regulated form of cell death distinct from apoptosis, is known to induce marked inflammatory responses and adaptive immunity effects in the body [103]. Previous studies have indicated that BBR may act as a necroptosis inducer in cancer cells, showcasing potential anti-tumor effects [104,105,106]. BBR usually plays a neuroprotective role in the treatments for neurological diseases and neuronal cells inhibiting necroptosis [107]. Ou et al. demonstrated that BBR treatment could significantly inhibit the expression of receptor-interacting protein 1 (*RIP1*) and *RIP3*, two key regulators of necroptosis, attenuating the neuroinflammatory response and cognitive impairment in the hippocampus tissues in rats exposed to excessive L-arginine [107].

**Figure 3:**
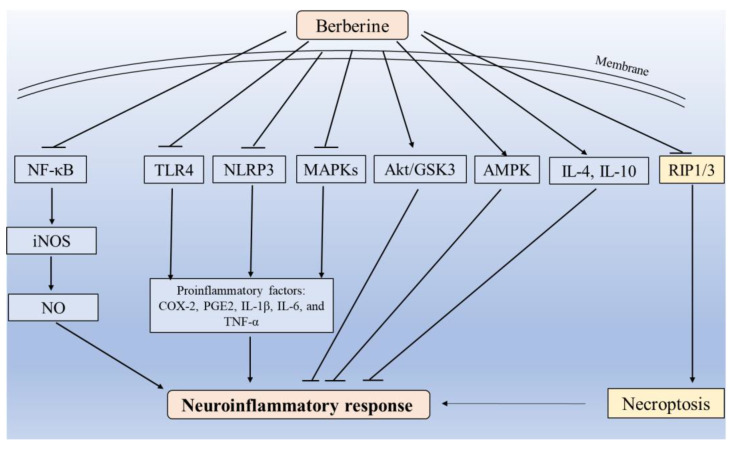
Proposed regulated mechanisms for BBR’s neuroprotection against neuroinflammatory response in nerve cells. NF-κB, nuclear factor kappa B; iNOS, inducible nitric oxide synthase; NO, nitric oxide; TLR4, toll-like receptor 4; NLRP3, NOD-like receptor protein 3; MAPK, mitogen-activated protein kinase; Akt, alpha serine/threonine-protein kinase; GSK3, glycogen synthase kinase 3; AMPK, AMP-activated protein kinase; IL-4, interleukin 4; IL-10, interleukin 10; RIP1/3, receptor-interacting protein 1/3; COX-2, cyclooxygenase 2; PGE2, prostaglandin E2; IL-1β, interleukin 1β; IL-6, interleukin 6; TNF-α, tumor necrosis factor-α.

In summary, the inhibitory effects of BBR on inflammatory response and necroptosis on nerve cells or tissues partly contributed to its neuroprotective properties. These effects are mediated through the inhibition of the MAPKs, AMPK, NF-κB, TLR4, and NLRP3 pathways, as well as direct modulation of inflammatory factor secretion, as illustrated in Figure 3.

### 3.3. Induction of Autophagy

Berberine (BBR) exhibits neuroprotective qualities by inducing autophagy and facilitating the clearance of toxic aggregate proteins [108,109,110]. BBR has been found to trigger autophagy in various cell types, including macrophages, lymphoblastic leukemia cells, retinal cells, and neuronal cells, as well as in various tissues such as the liver, lung, kidney, stomach, breast, and myocardium [111,112,113,114,115,116,117,118,119,120,121,122,123,124,125,126,127].

One of BBR’s mechanisms of neuroprotection involves promoting autophagy to eliminate misfolded proteins. Several studies have also found that BBR supplementation could effectively improve the motor dysfunction of mice with Huntingtin’s diseases via promoting the degradation of mutant Huntingtin protein through enhancing cell autophagy [9,109]. This process ultimately alleviates motor dysfunction and extends the survival period in a mouse model of Huntington’s disease [109]. Similarly, in an APP/tau/PS1 triple-transgenic mouse model of Alzheimer’s disease, BBR treatment could significantly improve the cognitive impairment of mice by promoting autophagy while inhibiting the production of β-amyloid (Aβ) through suppressing β-site APP cleavage enzyme 1 (*BACE1*) expression [110]. Moreover, in another study, data showed that BBR reduced the production of Aβ and the expression of the BACE1 protein by activating AMPK in nerve cells [128]. Interestingly, a great many studies have indicated that autophagy is AMPK-dependent or triggered via the AMPK/mTOR pathway [4,129]. Consequently, BBR may activate autophagy by the AMPK signal pathway to clear wrong proteins that cause neurological diseases.

**Figure 4:**
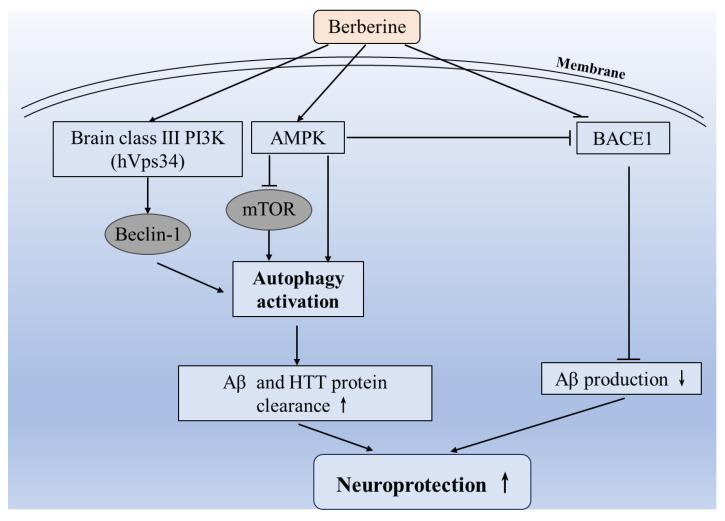
Proposed regulated mechanisms for BBR’s neuroprotection via the activation of autophagy. ↑ indicates the upregulation by BBR; ↓ indicates the downregulation by BBR. PI3K, phosphoinositide 3-kinase; hVps34, human vacuolar protein sorting 34; AMPK, AMP-activated protein kinase; mTOR, mammalian target of rapamycin; Aβ, β-amyloid; HTT, Huntington protein; BACE1, β-site APP cleavage enzyme 1.

Overall, a summary conferring the molecular mechanisms of BBR’s neuroprotection via inducing autophagy is shown in Figure 4.

### 3.4. Modulation of Neurotransmitters

Neurotransmitters are pivotal in facilitating intraneuronal communication and neurobehavioral functions [130]. BBR has demonstrated the ability to inhibit the activity of acetylcholinesterase, butyrylcholinesterase, and monoamine oxidases (MAOs), which play vital roles in regulating the levels of neurotransmitters [131,132]. For example, MAO-A and MAO-B, two isozymes of MAOs, are principally responsible for the degradation of various amine neurotransmitters, including DA, norepinephrine (NE), serotonin (5-HT), and epinephrine, all of which are crucial in the development of neurodegenerative diseases [133,134].

A previous study showed that oral administration of BBR at the final doses of 10 or 20 mg/kg could show a potent antidepressant-like effect in mice via upregulation of the levels of NA and 5-HT in the hippocampus and frontal cortex [131]. Additionally, BBR has been reported to significantly enhance DA levels in the brains of mice with Parkinson’s disease, attributed to increased gut Enterococcus abundance, thereby ameliorating Parkinson’s disease symptoms [37]. BBR administration has also been shown to suppress cholinesterase activity, thereby protecting the cholinergic system, and enhancing memory function in diabetic rats [67,133]. Glutamate is a primary excitatory neurotransmitter in the brains of animals and humans. N-methyl-d-aspartate (NMDA) is its receptor. Furthermore, BBR treatment has been found to protect against MK-801-induced neurodegeneration in rat brains by enhancing NMDA-mediated activity-dependent cell survival [135]. Similarly, BBR administration has been shown to significantly reduce the release of glutamate from rats’ cortical synaptosomes via inhibition of presynaptic Cav2.1 channels as well as the downregulation of the ERK/synapsin I signaling cascade [136].

**Figure 5:**
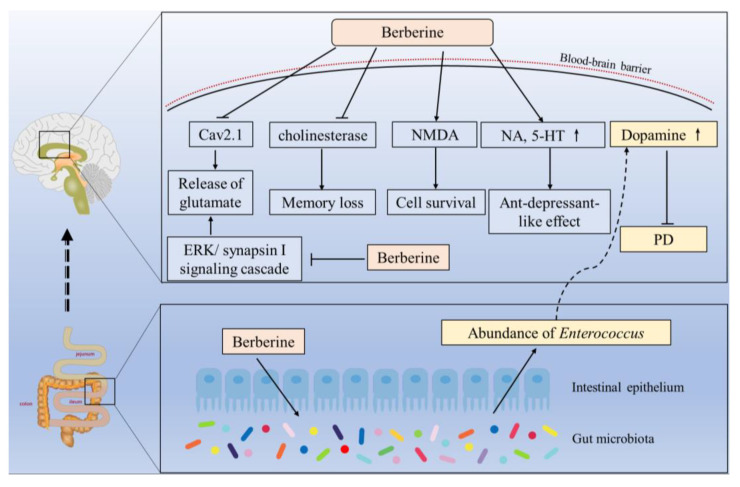
Proposed regulated mechanisms for BBR’s neuroprotection via the regulation of neurotransmitter levels in the brain. ↑ indicates the upregulation by BBR. Solid arrows indicate the direct effects and dashed arrows indicate the indirect effects. Cav2.1, voltage-gated Ca^2+^ channel 2.1; ERK, extracellular regulated protein kinases; NMDA, N-methyl-d-aspartate; NA, noradrenaline; 5-HT, 5-hydroxytryptamine; PD, Parkinson’s disease.

Overall, a summary conferring the molecular mechanisms of BBR’s neuroprotection via regulating neurotransmitter levels in the brain is shown in Figure 5.

### 3.5. Modulation of CYP450 Enzyme Activities

Recent studies have underscored the critical role that cytochrome P450 (CYP450) may play in the development or treatment of neurological diseases based on its effects on temperature control, maintenance of brain cholesterol homeostasis, neuropeptide release, and regulation of neurotransmitter levels [137]. It has been reported that BBR has a potent regulatory effect on CYP450 activities in liver, heart, kidney, and breast tissues [7,138,139,140,141,142,143,144,145,146,147].

Earlier investigations revealed that BBR could inhibit the activities of CYP1 enzymes, including CYP1A1, CYP1A2, and CYP1B1, with a stronger preference for CYP1B1 [148]. CYP1B1 has been reported to make an important contribution in the procession of various neurological disorders based on its regulated effects on the production of ROS and redox homeostasis [149]. It has been demonstrated that the deficiency of CYP1B1 could protect retinal astrocytes against oxidative stress and inflammation [150]. In addition, in a human clinical trial, researchers found that repeated administration of BBR (oral administration at a dose of 300 mg, three times daily, for 14 days) decreased CYP2D6, CYP2C9, and CYP3A4 activities [142,151]. Based on the inhibitory effects of BBR on the activities of the CYP3A4 enzyme, BBR significantly increases the blood concentration of cyclosporine when co-administrated [152]. In addition, p-glycoprotein (P-gp)-mediated gut efflux properties are responsible for the reduced bioavailability of BBR and verapamil, a p-gp inhibitor, and co-treatment could significantly enhance the neuroprotective effect of BBR against streptozotocin-induced cognitive dysfunction in a rat model [153].

To date, there is still limited information about the interaction between BBR and CYP450 enzymes in nerve tissue, and further investigations into the precise molecular mechanisms are warranted.

### 3.6. Others

Berberine (BBR) has demonstrated notable neuroprotective potential through various molecular pathways. Notably, it has been reported that BBR enhances synaptic plasticity by activating the cAMP response element-binding protein (CREB), leading to the production of brain-derived neurotrophic factor (BDNF) through the upregulation of *SIRT1*. This, in turn, contributes to the amelioration of cognitive impairment induced by chemotherapy drugs, such as doxorubicin [26]. In a transient middle cerebral artery occlusion rat model, BBR administration could induce gut–brain axis signal transmission and stimulate the vague nerve by inducing the production of intestinal hydrogen through the regulation of gut microbiota, finally offering neuroprotection [154]. Furthermore, BBR treatment has also been shown to reduce hippocampus neuronal damage by inhibiting the expression of the matrix metalloproteinase-9 protein and gelatinase activities and downregulating the expression of laminin and neuronal nuclei antigen (NeuN) proteins in the hippocampal CA1 and CA2 areas [155]. These functions also contribute to the therapeutic effects of BBR against multiple sclerosis disease [156]. Another study by Wu et al. highlights the neuroprotective potential of BBR by blocking neuronal ATP-sensitive K^+^ channels in substantia nigra pars compacta dopaminergic neurons [157]. BBR has also shown promise in enhancing nerve growth factor (NGF) expression, which promotes Nrf2- and Akt-related neurite outgrowth and differentiation, thus protecting against oxidative stress and neuroinflammatory responses [79,158,159].

## 4. Safety and Toxic Adverse of BBR

A series of studies has confirmed that the toxicity of BBR depends on the route and duration of administration [160,161,162,163]. Animal experiments showed that oral administration of BBR is safe and intravenous or intraperitoneal injection are toxic. For example, Kheir et al. showed that the median lethal dosages (LD_50_) of BBR via oral, intravenous, or intraperitoneal administration in mice are >20.8 g/kg, 9.04, and 57.6 mg/kg, respectively [160]. This difference is mainly dependent on the final concentration of BBR in the blood [160]. It also suggests that a single oral dose of BBR at 2.97 g/kg of body weight is safe according to the conversion of body surface area between humans and mice (approximately seven times between humans and mice) [164].

It has been reported that BBR exhibits potent cytotoxicity in vitro, exhibiting a potential anticancer effect [165]. BBR has been demonstrated to have potent nucleic-acid-binding activity, which is a key reason for inhibiting cell differentiation and inducing cycle arrest and DNA damage [166,167]. In addition, there are similar targets in the anticancer and neuroprotective effects of BBR, such as Akt, MAPKs, and Bcl-2 proteins, and the main difference are in the dosage [168]. Consistently, long-term administration and intraperitoneal or intravenous injection of berberine could induce multiple toxic effects, including neurotoxicity, immunotoxicity, phototoxicity, cardiotoxicity, and jaundice, and these toxic effects are in dose- and time-dependent manners [161,162,169]. This evidence also suggests that we may need safety measures with BBR, especially when administered intravenously or intraperitoneally in clinical practices. The toxic mechanisms of BBR may be related to its ability of directly interacting with DNA or inhibiting the presence of signaling pathways in cells [161,162,169,170].

In the clinic, it has been reported that oral administration of BBR at a dose of 1.5 g/day (500 mg per time for three times per day) for 13 weeks could result in potential gastrointestinal toxicity (such as diarrhea, constipation, flatulence, and abdominal complaint) in patients with type-2 diabetes, but no marked hepatotoxicity nor nephrotoxicity was detected [171].

Rad and colleagues systemically reviewed the toxic effects of BBR and *Berberis vulgaris* extract [161]. More information can be found in this review.

## 5. Clinical Trials and Therapeutic Applications

Accumulated evidence from clinical trials underscores the wide-ranging therapeutic applications of BBR. Among randomized clinical trials involving BBR and barberry (*Berberis vulgaris*) in the treatment of different human diseases, the effects of reducing lipids and improving insulin resistance are the most studied. Additionally, clinical investigations have delved into its potential benefits in cardiovascular, anticancer, gastrointestinal, central nervous system, and endocrine-related contexts. Importantly, oral administration of BBR has exhibited low toxicity and minimal side effects at standard doses, with occasional mild gastrointestinal reactions observed in some patients [10]. A randomized clinical trial involving 55 patients with acute ischemic stroke found that the effects of BBR combined with atorvastatin (20 mg/day) were better than that of atorvastatin alone (20 mg/day) [172]. In another clinical trial, oral administration of BBR at a dose of 1 g/day (i.e., 0.5 g per time, twice a day) for 16 weeks could effectively regulate the structure and function of the human gut microbiota, and *Bifidobacterium* probiotics could further enhance the hypoglycemic effect of BBR [173]. Li et al. found that oral BBR treatment could significantly reduce the serum intima–media thickness (IMT) and IL-6 levels, reducing the degree of carotid atherosclerosis to a certain extent and improving the neurological impairment and prognosis of acute cerebral ischemic stroke (AIS) patients [174]. In a recent study, oral administration of BBR at a dose of 0.5 g/day for 8 weeks significantly elevated blood DA levels in individuals with hyperlipidemia, suggesting a potential benefit in treating Parkinson’s disease [37]. While progress has been made in clinical trials exploring BBR’s potential for treating neurological diseases, further research remains imperative.

## 6. Conclusions and Future Directions

Currently, there are multiple studies indicating that oral BBR supplementation could provide potential neuroprotective effects against chronic neurodegenerative diseases or acute brain injury caused by ischemia–reperfusion or drugs. However, the oral bioavailability of BBR (less than 1%) is very limited [175]. It has been reported that the levels of BBR in brain tissues are lower than that in other tissues after oral administration [176]. More and more evidences suggest that gut microbiota may play an important role in various neurological diseases, and it is also the main mediator of BBR’s neuroprotective effect, but it still needs more clinical trials.

In conclusion, the published data indicate that the molecular mechanisms of BBR’s neuroprotective effects may involve multiple targets, including antioxidation, anti-inflammation, anti-apoptosis, anti-necroptosis, induction of autophagy, and modulation of CYP450 enzyme activities and gut microbiota. In addition, to overcome the poor bioavailability of BBR in clinical applications, recent advances in nanodrug delivery systems, encompassing polymeric-based, graphene-based, silver, lipid-based, dendrimer-based, magnetic mesoporous silica-based, and gold nanoparticles, have emerged as pivotal strategies. For example, nasal brain drug delivery technology, driven by nanotechnology, is gaining popularity in enhancing the efficacy of BBR in treating neurological diseases [177,178]. Future clinical studies are anticipated to elucidate the neuroprotective mechanisms of BBR further while optimizing its clinical efficacy and minimizing potential side effects. At this moment, while increasing the bioavailability of BBR, we may also need to consider its safety more. Furthermore, exploring the direct regulatory effects of BBR on CYP450 enzymes within the brain holds great clinical significance and represents an important avenue for research in uncovering the neuroprotective mechanisms of BBR.

## Data Availability

Not applicable.
